# Natal origin of Pacific bluefin tuna from the California Current Large Marine Ecosystem

**DOI:** 10.1098/rsbl.2019.0878

**Published:** 2020-02-05

**Authors:** R. J. David Wells, John A. Mohan, Heidi Dewar, Jay R. Rooker, Yosuke Tanaka, Owyn E. Snodgrass, Suzanne Kohin, Nathan R. Miller, Seiji Ohshimo

**Affiliations:** 1 Department of Marine Biology, Texas A&M University at Galveston, 1001 Texas Clipper Road, Galveston, TX 77553, USA; 2 Department of Wildlife and Fisheries Sciences, Texas A&M University, College Station, TX 77843, USA; 3 Southwest Fisheries Science Center, National Marine Fisheries Service, 8901 La Jolla Shores Drive, La Jolla, CA 92037, USA; 4 Pacific Bluefin Tuna Biology Group, Bluefin Tuna Resources Division, National Research Institute of Far Seas Fisheries, 5-7-1, Orido, Shimizu, Shizuoka 424-8633, Japan; 5 Jackson School of Geosciences, The University of Texas at Austin, 2275 Speedway Stop C9000, Austin, TX 78712, USA; 6 Fisheries Management and Oceanography Division, Seikai National Fisheries Research Institute, 1551-8 Taira-machi, Nagasaki 851-2213, Japan

**Keywords:** *Thunnus orientalis*, otolith chemistry, trace elements, migration, Pacific Ocean

## Abstract

Natal origin of subadult (age-1) Pacific bluefin tuna (PBT, *Thunnus orientalis*) from the California Current Large Marine Ecosystem (CCLME) was determined using natural tracers in ear stones (otoliths). Age-0 PBT collected from the two known spawning areas in the western Pacific Ocean (East China Sea, Sea of Japan) were used to establish baseline signatures from otolith cores over 4 years (2014–2017) based on a suite of trace elements (Li, Mg, Mn, Sr, Zn and Ba). Distinct chemical signatures existed in the otolith cores of age-0 PBT collected from the two spawning areas, with overall classification accuracy ranging 73–93% by year. Subadult PBT collected in the CCLME over the following 4 years (2015–2018) were then age-class matched to baselines using mixed-stock analysis. Natal origin of trans-Pacific migrants in the CCLME ranged 43–78% from the East China Sea and 22–57% from the Sea of Japan, highlighting the importance of both spawning areas for PBT in the CCLME. This study provides the first estimates on the natal origin of subadult PBT in this ecosystem using otolith chemistry and expands upon the application of these natural tracers for population connectivity studies for this species.

## 1. Introduction

Characterizing population dynamics and connectivity for highly migratory species remains challenging, especially for species that cross ocean basins. Pacific bluefin tuna (PBT, *Thunnus orientalis*) is widely distributed throughout the North Pacific Ocean and western South Pacific Ocean [1,2], although the dynamics within this range are not well understood and uncertainties about stock structure continue to complicate fisheries management [3]. PBT is managed under the assumption of a single stock in the Pacific Ocean with two discrete spawning areas in the western Pacific Ocean (WPO). One located around the Philippines north to the Ryukyu Islands incorporating both the Philippines Sea and East China Sea (hereafter: East China Sea, ECS) with spawning occurring from April to June and one in the Sea of Japan (SoJ) where spawning happens from July to August [4–7]. Age-0 PBT remain in waters around Japan, but at age 0.5–2 years, an unknown portion of the fish migrate east across the Pacific Ocean and enter the California Current Large Marine Ecosystem (CCLME) [1,8,9], where they remain for several years before returning to the WPO [10,11]. While the general pattern of these trans-Pacific migrations has been documented, questions remain about the origin of PBT in the CCLME and the contribution rates of recruits from the two spawning areas.

One tool that has been used across a range of species to examine population dynamics is otolith chemistry. The premise behind using otolith chemistry as a natural tag is that chemicals incorporated into the aragonite–protein matrix of the otolith are related to the physico-chemical conditions of the surrounding water mass, thus serving as a spawning area-specific signature [12,13]. Resorption or remobilization of deposited elements during ontogeny is negligible since otoliths are metabolically inert, and thus, these structures retain the elemental signatures over time leaving evidence of fish movements throughout their lives [9,14–16]. The approach has been used to source juvenile and adult Atlantic Bluefin Tuna (*Thunnus thynnus*) to spawning areas in the Atlantic Ocean [17,18] and also shows promise for determining the origin and movement of PBT [9,15].

For this study, we used natural chemical tags in PBT otoliths to identify the natal origin of individuals after their trans-Pacific migration to the CCLME. First, we examined chemical signatures for multiple cohorts of age-0 PBT from both spawning areas (ECS and SoJ) before they had migrated in order to obtain yearly baseline values for each spawning area. Next, core material of the otolith from subadult PBT in the CCLME was analysed to estimate the relative contribution of each spawning area. Here, we present the first predictions of the natal origin of PBT in the CCLME using otolith chemistry.

## 2. Material and methods

Age-0 PBT (less than 50 cm fork length (FL)) were collected over a 4-year period (2014–2017) from the ECS and SoJ through fisheries-dependent sampling by the National Research Institute of the Far Seas Fisheries, Japan. Within each spawning area, 10–20 individuals were sampled at multiple collection dates and locations during each year to obtain a representative baseline sample (table 1). Given differences in spawning times, hatch dates of age-0 PBT were determined by microstructure analysis of the second sagittal otolith to validate fish were born in the same area collected (Far Seas Lab 2014–2017, unpublished data). Subadult PBT (age-1, approx. 60–75 cm FL) were subsequently collected in the CCLME over the following 4 years (2015–2018) to age-class match to baseline samples. Subadult (age-1) PBT from the CCLME were collected through recreational fisheries across a range of locations and dates for each collection year (table 1).

**Table 1.** Summary statistics of PBT collected from both spawning areas (East China Sea, ECS, and Sea of Japan, SoJ) and CCLME. Sample size (*n*), mean size (cm FL) (±1 s.d.), size range (cm FL) and collection dates.

collection area	year	*n*	mean size	size range	collection dates
*age-0*					
East China Sea	2014	19	32.9 (13.2)	17.3–48.4	18 July 2014–26 Nov 2014
Sea of Japan	2014	10	27.3 (1.5)	25.2–29.4	26 Oct 2014–14 Nov 2014
East China Sea	2015	20	32.1 (12.5)	17.5–49.6	20 July 2015–14 Dec 2015
Sea of Japan	2015	10	27.3 (1.5)	25.3–29.2	23 Sep 2015–28 Oct 2015
East China Sea	2016	15	21.2 (1.47)	20.0–25.3	15 July 2016–1 Aug 2016
Sea of Japan	2016	15	23.7 (1.84)	21.0–27.3	16 Sep 2016–25 Sep 2016
East China Sea	2017	15	21.5 (0.96)	20.0–23.7	23 July 2017–25 July 2017
Sea of Japan	2017	15	22.6 (2.39)	20.1–27.2	14 Sep 2017–19 Sep 2017
*subadult (age-1)*					
CCLME	2015	40	71.9 (4.08)	61.6–76.7	17 Jan 2015–20 Sep 2015
CCLME	2016	40	69.7 (4.07)	60.6–75.7	10 June 2016–30 Aug 2016
CCLME	2017	40	68.5 (3.97)	57.2–75.8	2 May 2017–28 Aug 2017
CCLME	2018	40	64.6 (3.12)	56.2–68.3	28 June 2018–29 Sep 2018

Following extraction and cleaning, sagittal otoliths were embedded in Struers EpoFix resin and sectioned using a low-speed diamond blade saw to obtain a 1.5 mm transverse section that included the otolith core. Otolith sections were polished to the core and then attached to a sample plate using Crystalbond™ thermoplastic glue. Elemental concentrations were quantified using an Elemental Scientific NWR193UC (193 nm wavelength, less than 4 ns pulse width) laser ablation system coupled to an Agilent 7500ce inductively coupled plasma mass spectrometer (LA-ICPMS) at the University of Texas at Austin. Prior to analysis, samples and standards were pre-ablated (60% laser energy, 20 Hz, 75 µm spot, 2 s dwell) to remove potential surface contamination. Ablation parameters included 60% laser energy, 10 Hz, 50 µm spot, 30 s dwell and unknown samples were bracketed hourly by standard measurements on the LA-ICPMS (MACS-3 and NIST 612, typically measured in triplicate for 60 s). Laser energy densities over the analytical sessions averaged 2.97 ± 0.03 J cm^−2^ for spot analyses. The quadrupole time-resolved method measured 13 masses using integration times of 10 ms (^24^Mg, ^43–44^Ca, ^88^Sr, ^115^In), 20 ms (^25^Mg, ^55^Mn) and 50 ms (^7^Li, ^59^Co, ^63^Cu, ^66^Zn, ^137–138^Ba). Time-resolved intensities were converted to concentration (ppm) equivalents using Iolite software, with ^43^Ca as the internal standard and a Ca index value of 38.3 weight %. USGS MACS-3 was used as the primary reference standard and accuracy and precision were proxied from replicates of NIST 612 analysed as an unknown. NIST 612 analyte recoveries (*N* = 72) were typically within 2% of GeoREM preferred values (http://georem.mpch-mainz.gwdg.de). To characterize the core signature, the average was taken of five ablation spots, the first at the otolith core (defined as the narrowest part of the section), followed by two equally spaced spots on each side of the core [19] corresponding to approximately the first month of life based on previous estimates of otolith accretion rates.

Multiple statistical approaches were used to compare otoliths from the two spawning areas and the CCLME. Two-way mixed-effects analysis of variance (ANOVA) was used to test for differences in element : Ca ratios in the otolith cores of age-0 PBT with year, spawning area and year × spawning area interaction as fixed factors. Principal components analysis was used to examine the relationship of each element : Ca ratio to samples collected between the two spawning areas. Quadratic discriminant function analysis (QDFA) was used to determine the classification accuracy of PBT to each spawning area based on the jackknife reclassification. Estimates of nursery origin for PBT collected from CCLME were derived by comparing element : Ca ratios in the otolith cores of age-1 PBT to the corresponding baseline data of age-0 PBT. Natal origin (ECS versus SoJ) of subadult PBT collected in the CCLME was predicted using the maximum-likelihood mixed-stock analysis program HISEA [20]. Mixed-stock analyses included bootstrapping with 1000 simulations to obtain estimates of uncertainty (error terms) [20]. Statistical significance for all tests was set at the *α*-level of 0.05.

## 3. Results

Otoliths from 119 age-0 PBT (*ca* 30 per year) collected from 2014 to 2017 were analysed to establish baseline signatures for each spawning area (table 1). Sizes of age-0 PBT ranged from 17 to 49 cm FL with an average size of 26.4 cm FL (±8.6 s.d.). Age-0 PBT from the two spawning areas were similar in size averaging 29.5 cm FL (±11.9 s.d.) and 24.1 cm FL (±2.8 s.d.) in the ECS and SoJ, respectively (table 1). Age-1 PBT collected from 2015 to 2018 in the CCLME (40 per year) ranged in length from 56.2 to 76.7 cm FL with an average size of 68.7 cm FL (±4.6 s.d.) (table 1).

Element : Ca ratios in otolith cores of age-0 PBT significantly differed both between ECS and SoJ spawning areas and among years (*p* < 0.01) (electronic supplementary material, figure S1). Significant interactions between spawning area and year highlight the necessity of obtaining element : Ca baselines for age-0 PBT each year from both the ECS and SoJ. Mg : Ca, Mn : Ca and Sr : Ca were the most influential elements and significantly different between spawning areas in multiple years, although no element : Ca was significantly different between the two spawning areas in all years. Mg : Ca was significantly higher within otolith cores of age-0 PBT from the ECS in 2014 (Tukey HSD, *p* < 0.05), but significantly lower in both 2015 (*p* < 0.01) and 2016 (*p* < 0.001) relative to the SoJ. Mn : Ca was significantly higher in otolith cores from the SoJ during 2014 (*p* < 0.001) and 2017 (*p* < 0.01) but did not differ in 2015 or 2016. Sr : Ca was significantly higher in otolith cores from the ECS only in 2014 (*p* < 0.001). Li : Ca did not differ between spawning areas (*p* > 0.05) but was significantly higher in 2016 and 2017 than in 2014 and 2015 (Tukey HSD, *p* < 0.001). No differences were detected for Zn : Ca or Ba : Ca between spawning areas or across collection years.

**Figure 1.**
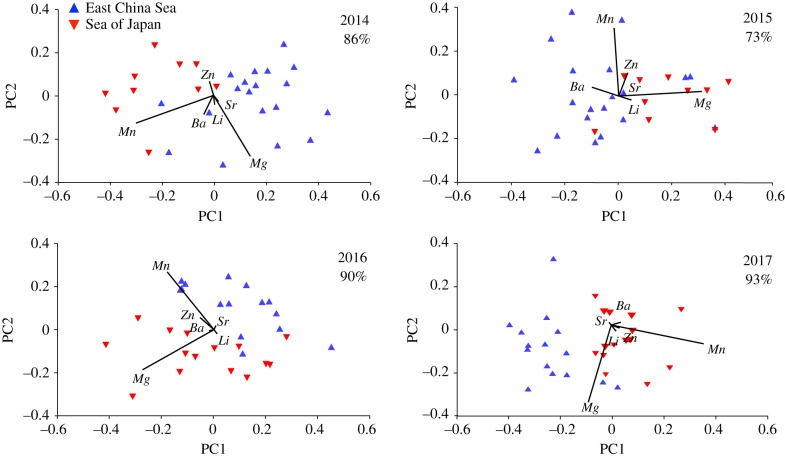
PCA plot of otolith core chemistry from age-0 PBT in both spawning areas, East China Sea (blue) and Sea of Japan (red). Elemental ratios used for PCA included Ba : Ca, Li : Ca, Mg : Ca, Mn : Ca, Sr : Ca, Zn : Ca. (Online version in colour.)

Classification success of age-0 PBT to the ECS or SoJ combined across years was 76%. Results differed by year with classification success of 86% in 2014, 73% in 2015, 90% in 2016 and 93% in 2017 (figure 1). Otolith element : Ca ratios varied by year, with Mg : Ca and Mn : Ca most important for classification between spawning areas. Principal component analysis (PCA) axes 1 and 2 combined for 79.2% (2016) to 86.4% (2017) of the cumulative variance explained with Mg : Ca and Mn : Ca both having the highest PCA coefficients for each axis during all years (figure 1).

**Figure 2.**
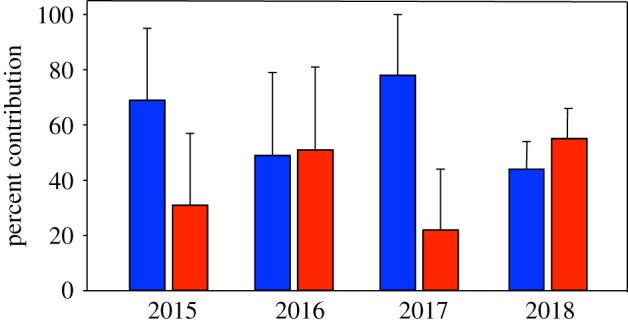
Natal origin contribution estimates (mean ± s.d. percent contribution) of subadult (age-1) PBT collected in the CCLME.

Mixed-stock analysis of age-1 PBT collected in the CCLME indicates that migrants from both the ECS and SoJ recruited into the eastern Pacific Ocean (figure 2). Age-class matched otolith cores of age-1 PBT to the age-0 baseline showed that contribution rates varied from year to year, with both spawning areas contributing significant numbers of recruits to the CCLME (figure 2). Age-1 PBT collected in 2015 and 2017 from the CCLME were primarily from the ECS spawning area with estimates of 69% and 78%, respectively. By contrast, the majority of subadults in 2016 and 2018 were from the SoJ spawning areas (51% and 57%, respectively).

## 4. Discussion

Results highlight the importance of both the ECS and SoJ spawning areas to the PBT fishery in the CCLME. In the western Pacific, there have been multiple efforts to identify natal origin, all capitalizing on the difference in spawning time between the two spawning areas. Itoh [21] assigned natal spawning areas by back calculating the hatch date from size and daily rings in otoliths; however, this approach was only valid at smaller age classes. Shiao *et al*. [6] measured oxygen isotopes from the otolith core to the margin, which became enriched as temperatures cooled in the winter and resulted in an earlier increase in otolith oxygen values for fish spawned in the SoJ relative to ECS. Similarly, Uematsu *et al*. [22] measured the vertebral distance from the vertebrae focus to the first annulus, which formed during winter and found this distance was shorter for fish spawned later in the year from the SoJ. Rooker *et al*. [15] demonstrated the potential for using chemistry of whole otoliths to compare nursery areas, but whole otolith approaches integrate complete life-time signatures. Our study provides a 4-year assessment sourcing the natal origin of recruits in the eastern Pacific Ocean using laser ablation with high spatial resolution to sample only the early-life period and supports the utility of the approach for future expansion of multiple age classes throughout the Pacific Ocean. The longer and earlier spawning season in the ECS spawning area relative to the SoJ may contribute to more recruits in the western Pacific [21]; however, this pattern was only found in two of the four years in the CCLME. Given that growth and survival of PBT are related to sea surface temperature (SST) and prey availability at the early-life stage [23,24], future research should examine relationships between early-life survival and environmental conditions within each spawning area relative to recruitment into the CCLME.

Interannual variability observed in element : Ca ratios emphasizes the need to have annual baseline samples of age-0 PBT collected from both spawning areas to enable age-class matching. In the present study, Mg : Ca, Mn : Ca and Sr : Ca were most important in distinguishing between the two spawning areas, although annual variability of each element was significant. Similarly, Rooker *et al*. [15] found significant interannual variability in trace elements of whole otoliths from age-0 PBT, including Mg : Ca and Mn : Ca, the two most useful tracers in this study. Several factors can influence the uptake of chemicals into the otolith aragonite matrix including physiology, temperature, diet and water chemistry [13]; however, isolating the driving factors are out of the scope of this study. Interannual differences in riverine input and upwelling intensity may partly explain the elemental fluctuations due to the proximity of the spawning areas to land and river interface [15]. Other potential factors include large-scale climactic conditions such as El Nino Southern Oscillation (ENSO) and Pacific Decadal Oscillation (PDO) which affects precipitation, SST and sea surface salinity. Elements such as Mg and Mn may also be under physiological control and related to metabolic activity [25,26]. Differences in SST during spawning may lead to varying metabolic rates between the spawning areas, resulting in variable otolith Mn : Ca and Mg : Ca. SST are 3°C warmer in the ECS (28°C) relative to the SoJ (25°C) during relative spawning seasons [5]. Thus, variation in fish physiology, mediated by temperature and prey sources, likely contribute to the elemental differences detected in PBT otoliths between the spawning areas.

In addition to natal origin, multiple studies have examined movements of PBT throughout the Pacific. Annual recruitment and the timing of trans-Pacific migrations vary as a function of SST, currents and food availability [27–29]. Fujioka *et al*. [28,29] used archival electronic tags to examine the movement patterns of age-0 PBT in the western Pacific and suggested cold waters (less than 14°C) may trigger this approximately 8000 km trans-Pacific migration to the CCLME. Oceanographic structures such as warm-core eddies and dynamics of the Kuroshio–Oyashio current system also affect the timing and movement of individuals [28,29]. Recent studies also suggest spawning may occur near or within the Kuroshio–Oyashio transition area [30,31], which warrants further research. In a separate study, Madigan *et al*. [11] used stable isotopes in muscle combined with radiocesium to infer movement of PBT and found fish may spend 2–5 years in the eastern Pacific before returning to the western Pacific. This study adds to the tools available to examine movements of PBT. Ultimately, a combination of approaches needs to be explored to understand the overall connectivity and movement dynamics of this valuable species within the Pacific Ocean basin, and this study provides the first predictions of the natal origin of PBT in the CCLME using otolith chemistry. Additional years of analyses are needed to characterize the patterns in recruitment to the CCLME and how variability may be linked to larval recruitment and conditions in the western Pacific.

## Supplementary Material

Supplementary Figures

## Data accessibility

The data supporting this article are available at the Dryad Digital Repository: https://doi.org/10.5061/dryad.cnp5hqc1k [32].
