# Supplementary figures and images for: Natal origin of Pacific bluefin tuna from the California Current Large Marine Ecosystem
Source: Biol Lett. 2020 Feb 5;16(2):20190878. doi: 10.1098/rsbl.2019.0878 (PMC7058956; doi:10.1098/rsbl.2019.0878)

## Slide 1
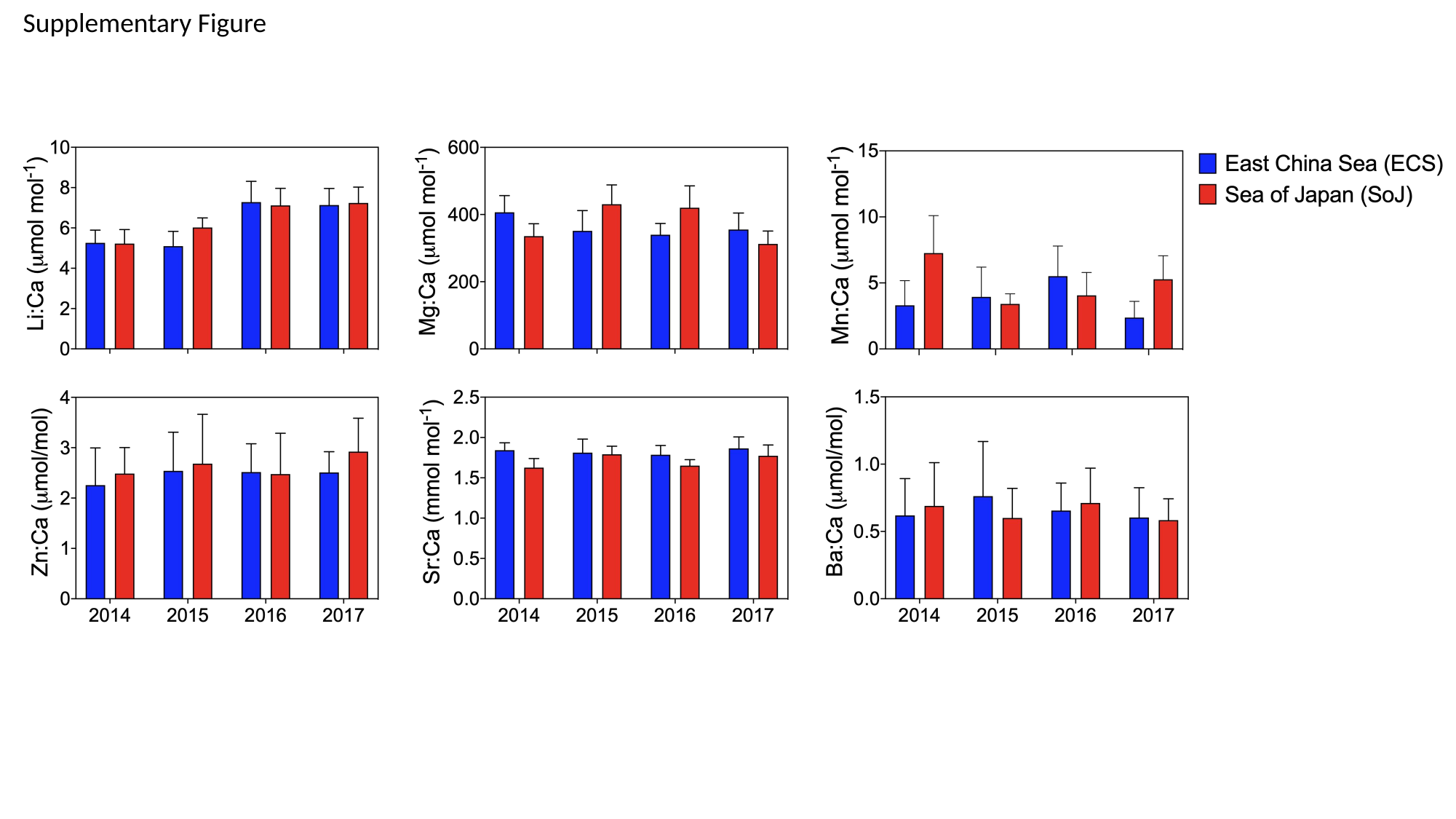

Supplementary Figure

Supplement: Supplementary Figures [file rsbl20190878supp1.pptx]
